# Understanding Participant-Centered Recruitment in Aging Research Through Decision Making

**DOI:** 10.1093/geroni/igaf122.2759

**Published:** 2025-12-31

**Authors:** Susan Passmore, Faith Ocoko, Madisin Randolph, Morgan Medina, Carey Gleason

**Affiliations:** University of Wisconsin–Madison, Madison, Wisconsin, United States; University of Wisconsin–Madison, Madison, Wisconsin, United States; University of Wisconsin–Madison, Madison, Wisconsin, United States; University of Wisconsin–Madison, Madison, Wisconsin, United States; University of Wisconsin–Madison, Madison, Wisconsin, United States

## Abstract

Despite decades of evidence of the problem, participant samples in aging research often fail to adequately represent the population of older adults. For example, clinical research studies of Alzheimer’s Disease, which disproportionately affects African American and Latine adults, frequently include over-representations of non-Hispanic White participants, limiting our full understanding of disease presentation and our ability to provide appropriate care for patients and caregivers. Authentically participant-centered research engagement offers promise to address this problem. However, few studies have specifically addressed how to create studies that participants, particularly those who are underrepresented currently, find acceptable and accessible. This presentation will include findings from a study of the real-world decision-making processes for African American adults (aged 53-77 years old) who had recently decided on their willingness to participate in the African Americans Fighting Alzheimer’s in Midlife (AA-FAIM) study. Semi-structured interviews were conducted with those who eventually agreed to participate in AA-FAIM (n = 25) and those who declined participation (n = 18). Interviews were recorded and transcribed to facilitate a thematic analysis following Braun and Clarke. Findings reveal both positive and negative factors weighed in decision-making, including study team interactions, past experiences in research, personal experiences with Alzheimer’s Disease, study requirements, and accessibility. Participants also offered that relationships with other study participants, a factor not reported in similar studies, figured into decision-making for many of those we interviewed.

